# Automated alternate cover test for ‘HINTS’ assessment: a validation study

**DOI:** 10.1007/s00405-021-06998-w

**Published:** 2021-07-23

**Authors:** Miranda Morrison, Hassen Kerkeni, Athanasia Korda, Simone Räss, Marco D. Caversaccio, Mathias Abegg, Erich Schneider, Georgios Mantokoudis

**Affiliations:** 1 Department of Otorhinolaryngology, Head and Neck Surgery, Inselspital, University Hospital Bern and University of Bern, 3010 Bern, Switzerland; 2 Department of Neurology, Inselspital, Bern University Hospital, University of Bern, Bern, Switzerland; 3 Department of Ophthalmology, Inselspital, Bern University Hospital, University of Bern, Bern, Switzerland; 4 Brandenburg University of Technology Cottbus, Senftenberg, Germany

**Keywords:** Skew deviation, Validation, Test of skew, Alternate cover test, Video-oculography

## Abstract

**Objective:**

The alternate cover test (ACT) in patients with acute vestibular syndrome is part of the ‘HINTS’ battery test. Although quantitative, the ACT is highly dependent on the examiner’s experience and could theoretically vary greatly between examiners. In this study, we sought to validate an automated video-oculography (VOG) system based on eye tracking and dedicated glasses.

**Methods:**

We artificially induced a vertical strabismus to simulate a skew deviation on ten healthy subjects, aged from 26 to 66, using different press-on Fresnel prisms on one eye while recording eye position with VOG of the contralateral eye. We then compared the system’s performance to that of a blinded trained orthoptist using conventional, semi-quantitative method of skew measurement known as the alternate prism cover test (APCT) as a gold standard.

**Results:**

We found a significant correlation between the reference APCT and the Skew VOG (Pearson’s *R*^2^ = 0.606, *p* < 0.05). There was a good agreement between the two tests (intraclass correlation coefficient 0.852, 95 CI 0.728–0.917, *p* < 0.001). The overall accuracy of the VOG was estimated at 80.53% with an error rate of 19.46%. There was no significant difference in VOG skew estimations compared with the gold standard except for very small skews.

**Conclusions:**

VOG offers an objective and quantitative skew measurement and proved to be accurate in measuring vertical eye misalignment compared to the ACT with prisms. Precision was moderate, which mandates a sufficient number of tests per subject.

**Supplementary Information:**

The online version contains supplementary material available at 10.1007/s00405-021-06998-w.

## Introduction

Vertical misalignment of the eyes is considered a red flag in acute dizziness as it may indicate presence of a vestibular stroke, in which case, it is termed skew deviation. Skew deviation can occur in patients with acute vestibular tone imbalance and is often part of the ocular tilt reaction (OTR). Its origin is derived from lesions of the graviceptive pathways leading to a classical trias of head tilt, skew deviation and ocular counter-roll [4]. OTR was first observed in animal models by Magendie–Hertwig in 1833 and 1855 [5]. The graviceptive pathways including otolithic and vertical semicircular canal pathways are responsible for postural stability but also for gaze stability during translational movements as well as head rotation and tilt in the roll plane. Peripheral lesions might also induce skew deviation if there is a complete vestibular loss; [6–8] however, skew in vestibular strokes is believed to be larger and more sustained [9]. The eye is lower on the ipsilateral side in peripheral lesions or pontomedullary lesions; however, central lesions at higher levels lead to a contraversive reaction since the graviceptive pathways are crossing to the contralateral side at the level of the pontomedullary junction. Vertical eye misalignement can also occur with many other lesions such as trochlear palsy [10].

Skew deviations can be clinically assessed either qualitatively by the alternate cover test (ACT) [1] or quantitatively by the additional application of prisms in combination with the ACT [1]. The alternate prism cover test (APCT), however, relies strongly on the experience of the examiner [2]. Currently there are no validated objective, quantitative tests of skew available in the emergency department (ED). Previously described semi-automated methods for skew measurements were suitable for neuro-ophtalmology laboratories and customized for the assessment of strabismus [3].

We sought to test healthy subjects under standard conditions with quantitative recording of eye movements. The test of skew was assessed quantitatively by recording vertical eye positions with a portable video-oculography (VOG) device suitable for the assessment of vestibular reflexes in the ED. The diagnostic accuracy of such an eye-tracking device measuring vertical misalignment of the eyes (skew deviation) is unknown.

To test the validity of VOG in detecting skew, we artificially induced skew deviation on healthy subjects using different self-adhesive Fresnel prism (FP) foils on one eye while recording eye position with VOG. We then compared the system’s performance to that of a trained orthoptist using the conventional, semi-quantitative method of skew measurement (APCT) as a gold standard.

## Methods

We artificially induced skew in 10 healthy participants aged from 26 to 66 (mean 39.0 y ± 11 y, 3 males, 7 females) using FP on the left eye: we then undertook the VOG and the APCT measurements. The refractory value of the FP ranged from 1 to 10 prism diopters (PD) [1 PD (0.57°), 2 PD (1.14°), 4 PD(2.28°), 6 PD (3.42°), 8 PD(4.56°) and 10 PD (5.70°)]: these were randomly applied (random order derangement). The examiner of the APCT was blinded regarding the FP used, but could choose between different prism strengths ranging from 1 to 10 PD. We compared VOG results to the actual value of the FPs and correlated the obtained results with the current gold standard of the ACPT. We excluded all subjects (1) having previously experienced dizziness, (2) suffering from a previous neurological disorder (including cognitive impairment), (3) suffering from poor vision, (4) double vision, (5) strabismus and (6) any pregnant subjects.

### VOG setup

**Fig. 1:**
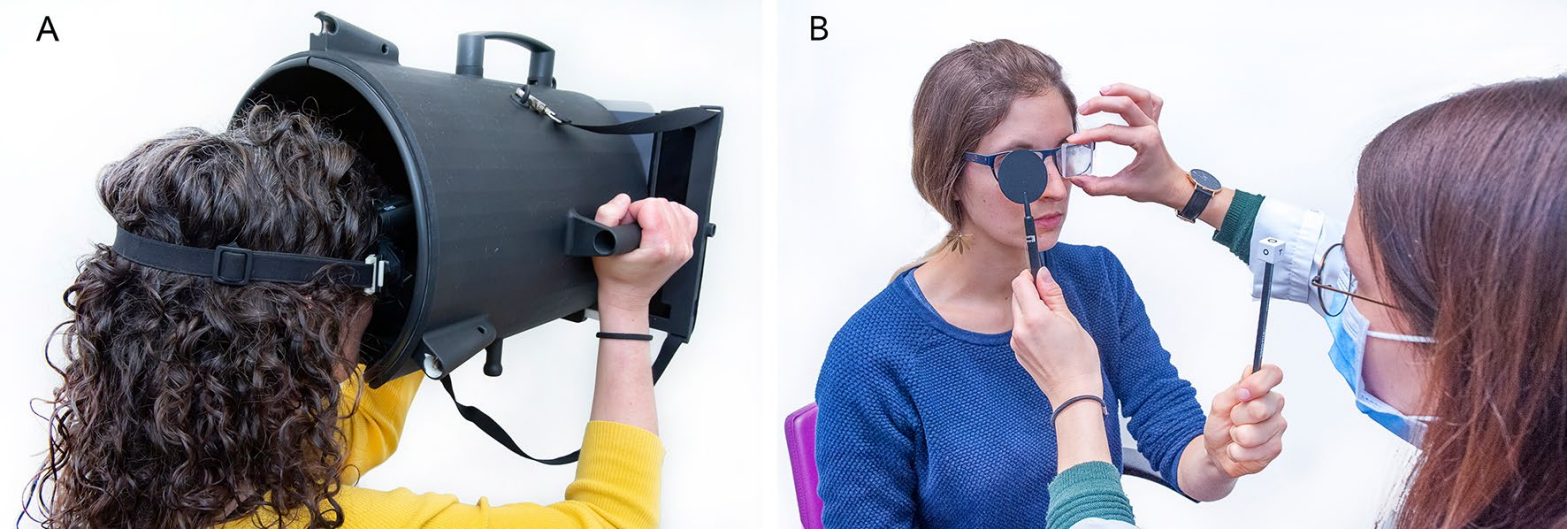
Panel **A**. The Skew VOG-Test is a portable device consisting of a tablet screen that is mounted on the back of a bucket. The subject is equipped with VOG googles for measuring eye position using pupil-tracking software. Panel **B**. APCT: a trained orthoptist estimated the induced skew performing the alternate cover test with prisms opposed to the subjects left eye. The prisms were changed in diopter size until there was no corrective vertical eye movement during the alternate prism cover test

We used a fixation target (4 mm diameter) at 260 mm distance (0.881° relative to patient view) on a tablet screen (luminosity = 6.17 Lux). The distance to the tablet was constantly maintained by a custom bucket with a chin rest inside and the tablet screen at the bottom of the bucket (Fig. 1A). The subject had to hold their heads in the bucket on the chin rest and wore VOG goggles (EyeSeeCam^™^, Munich) with a high-speed infrared camera mounted on the goggles frame for right eye positional tracking. We used color-filtered glasses on both eyes (red filter for left eye and blue filter for right eye). For each eye, the color filters allowed only monochromatic view of the target, which changed periodically (2 s intervals) from red to blue and vice versa. VOG goggles were branched by USB cable to a laptop and the right eye position was recorded after a calibration procedure using five targets on the tablet (center, vertical and horizontal  ± 8.5°). The timing of the target display on the tablet was synchronized with VOG using WiFi synchronization signals. Synchronization was mandatory for an accurate analysis of skew. The skew values were calculated by detecting the saccades and computing the area under the velocity curve between the start of the first saccade and the end of the last within the event window (−0.5–1.5 s around LED switch time).

Subjects were asked to maintain their eyes closed until recording started. We then performed a 10 s long measurement with minimum of five trials (target color change) per FP foil.

### Alternate cover test with prisms

All subjects underwent a second set of measurements, after a washing out period of 2 min, during which a trained orthoptist estimated the artificially induced skew using a system of refractory prisms opposed to the subjects left eye and the ACT (Fig. 1B) in a bright room. The order of tests (orthoptist versus VOG) was randomly deranged to avoid a potential order bias.

### Statistical analysis

We performed a descriptive statistic including the measurement of precision, variance and accuracy of VOG (% error  =  (alternate cover test−VOG)/alternate cover test × 100%). We measured the correlation between conditions calculating the Pearson’s *R* coefficient. We calculated the reliability between the two tests using the intraclass correlation coefficient for continuous data. We compared the mean value of the two tests in each condition using a two-sided, paired, non-parametric “Wilcoxon test”. We used SPSS for the statistical analysis (IBM SPSS Statistics for Windows, Version 25.0. Armonk, NY: IBM Corp.).

## Results

**Fig. 2:**
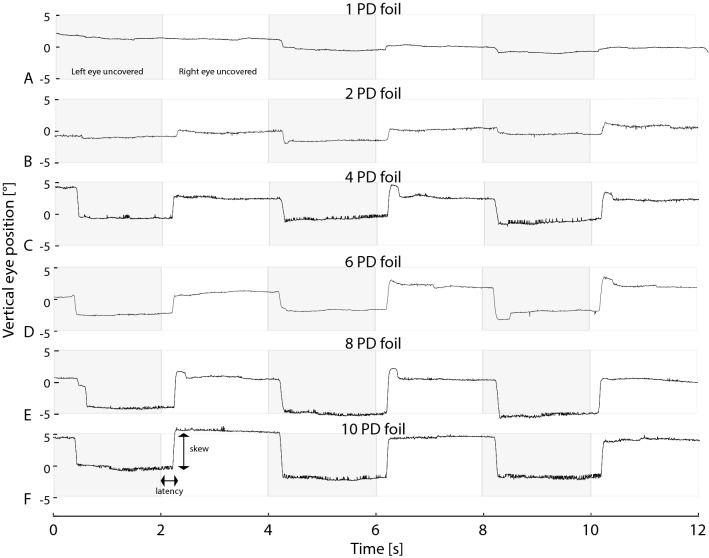
Vertical positions of the right eye over time are shown with black lines. Periods of right and left eye occlusions are plotted in front of white and grey backgrounds, respectively. Curves **A**–**F** show increasing foil strengths, respectively [**A** 1 PD foil (0.57°), **B** 2 PD foil (1.14°) **C** 4 PD foil (2.28°) **D** 6 PD foil (3.42°) **E** 8 PD foil (4.56°) and **F** 10 PD foil (5.70°)]. Please note, that hypermetric saccades have not been included by the VOG algorithm

Quantitative measurements of skew (with the VOG device) were feasible in all participants. The average examination time was 15 min including the calibration procedure. Figure 2 shows an example of positional data recorded with VOG at various conditions. We observed a corrective saccade either up or downwards with a short latency after the change of eye viewing regardless of the prism size. However, three participants did not show any saccadic eye corrections with FP size of 1 PD (0.57°) and 3 had no eye alignment change with FP sizes of 2 PD (1.14°).

The mean value of the measurements was not significantly different between the APCT and the VOG except for two conditions [1 PD (0.57°) and 4 PD (2.28°)]. In the 1 PD (0.57°) condition the mean value in the APCT measurements was overestimated at 3.90 ± 2.88 PD (2.22  ± 1.64°) and for the VOG was 1.30 ± 1.83 PD (0.74 ± 1.04°) (*p* = 0.09). In the 4 PD (2.28°) condition the mean value in the APCT measurement was 3.50 ± 1.43 PD (1.99 ± 0.81°) and for the VOG was 2.14 ± 1.69 PD (1.21 ± 0.96°) (*p* = 0.28). The detailed results of the mean values for the APCT and the VOG are summarized in Table S1 (supplementary material).

**Fig. 3:**
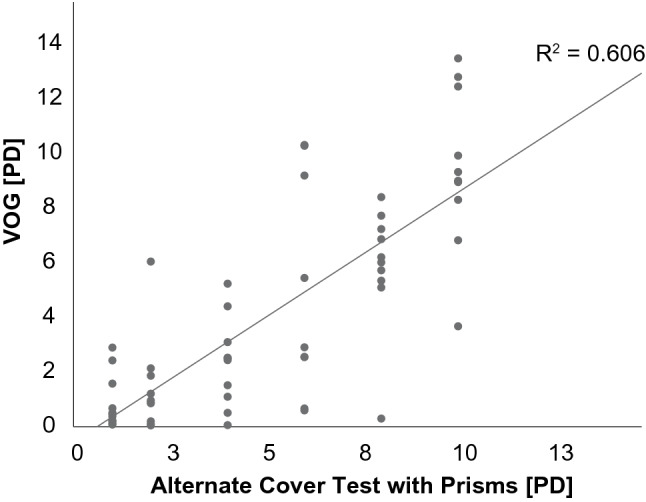
Correlation between the alternate cover test with prisms (performed by an orthoptist) and VOG device (Pearson’s *R*^2^ = 0.606, *p* < 0.05)

**Fig. 4:**
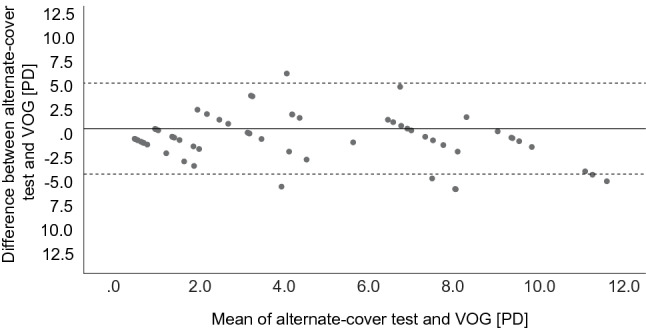
Bland–Altman plot: analysis of the agreement between the APCT and the VOG. The difference between APCT and the VOG plotted against the mean value for both the ACT and the VOG. The solid line shows the mean of the differences, while the dashed lines represent the superior and inferior limits of agreement

We found a significant correlation between the reference APCT and the Skew -VOG (Fig. 3A, Pearson’s *R*^2^ of 0.606, *p* < 0.05). There was a good agreement between the two tests (intraclass correlation coefficient 0.852, 95 CI 0.728–0.917, *p* < 0.001). The overall accuracy of the VOG was estimated at 80.53% with an error rate of 19.46%. We performed a Bland–Altman plot (Fig. 4), which demonstrated consistent variability across the graph with no proportional bias with a mean value of 1.8 PD (1.02°) and a half width of the 95% limit of agreement of  ± 5.2 PD (2.96°).

Figure S1 (supplementary material) shows the variance of both measurement methods (APCT and VOG) as a marker for test precision. There was a high variance across conditions ranging from 0.7 to 6.9 PD (0.40–3.93°). For conditions with small foil values [1 PD (0.57°)] the variance of APCT was markedly higher [*s*^2^ = 7.7PD (4.39°)] than Skew VOG [*s*^2^ = 3.5 PD^2^ (1.99°)].

## Discussion

The Skew VOG test is an easy to perform and reliable instrument for the measurement of Skew Deviation. We found that the system was accurate and precise in its detection of artificially induced skew deviation. Our results demonstrated a good correlation and agreement between the values measured by the Skew VOG-Test and the reference gold standard (APCT).

### Current automated system models

A number of automated systems for the measurement of eye misalignment have been described in the literature, and they have mainly been used for screening or detecting strabismus in adults or children [3]. Different eye-tracker systems allow for the accurate detection of eye position [11]. Although good for detecting the latent components of strabismus they cannot be used in patients with nystagmus or high refractive error (nanophtalmos or pathological myopia) [12]. Binocular optical coherence tomography calculates eye position using the measurement of corneal vertex reflection in the fixating eye (draws line between pupil margins at the posterior epithelium of the iris of both eyes). This allows for the detection of subtle differences in the size of the strabismus, not visible to the naked eye, it is, however, incapable of differentiating between heterophoria and heterotropia [13]. Finally, some systems simply use a camera mounted directly in front of the patient on a laptop linked to eye-tracking image analysis software to film the eyes. The advantage of such a setup is its portability and ease of use. It is, however, incapable of measuring the exact angle of the strabismus [14] and such an integrated system would not be convenient for bedside testing.

To test for skew, the eyes have to be occluded in an alternating manner, this can be done either manually or automatically. Some systems such as the video-Goggle Hess Screen Test use an automated LCD shutter, which occludes the vision in an alternate manner between the left and right eye, thus allowing for the measurement, if needed, of latent strabismus and skew [15]. Other semi-automated systems need a manual mouse/trigger indicating the alternating frequency [16]. Our device (EyeSeeCam^™^), however, used a synchronized trigger on a tablet system that provided an automated alternating color stimulus to allow monocular alternating vision by color filters. Eye-tracking systems adapted for vestibular testing such as the GN Otometrics^™^ or the EyeSeeCam^™^ systems use goggles with a single, high-speed, infrared camera [16]. These multipurpose devices allow a comprehensive and integrated battery for bedside vestibular testing including static and dynamic vestibular tests.

### Accuracy and precision of VOG skew testing

We found a good correlation between VOG and the orthoptist examination, which is in line with previous studies [3]. There was no significant difference of skew measurements across all conditions except 1 PD (0.57°) and 4 PD (2.28°). Small skews were significantly overestimated by the orthoptist. VOG showed a good accuracy and was not inferior to APCT. The VOG system offers a better precision (small standard deviation) for little skew deviations [1−2 PD (0.57−1.14°)]; however, the overall precision was moderate due to small eye-tracking artifacts. This finding mandates a sufficient number of tests per subject.

### The advantage of VOG skew testing

Thanks to their ease of use and reproducibility, automated systems do not rely on expert knowledge and can thus be used by non-specialists reliably. Although the APCT is a fast and effective clinical exam for the semi-quantitative detection of eye misalignment, it has the disadvantage of being highly dependent on the examiner’s experience, [5] thus leading to high interrater variability [5].

Such clinical tests are also time consuming and the patients’ cooperation might be limited because of dizziness and nausea. Experts, familiar with eye movement examinations, however, might not benefit as much as non-experts from an automated system, unless the automated skew test is part of a comprehensive battery of other vestibular tests integrated into one and the same device like the one we evaluated here. There are other clinical signs associated with skew deviation in dizzy patients such as ocular counter-roll detected by fundoscopy, [19] subjective visual vertical, [17] the maddox rods, [18, 19] or by the Hess–Lancaster test and Parks–Bielschowsky test [19]. Such additional tests performed by experts could add more diagnostic certainty in patients with suspected skew deviation.

### Strengths and limitations

This is the first study reporting the diagnostic accuracy of automated skew deviation measurements by a portable VOG system designed for the assessment of dizzy patients in the ED. We did not test, however, the specificity of the VOG device in measuring misalignments in subjects without inducing any skew. Unlike the APCT, it allows a precise quantification of eye misalignment with an objective measure of eye excursion.

Our investigative setup is not generalizable to all other systems on the market. We used VOG goggles, designed and approved for measuring static and dynamic vestibular reflexes such as the VOR. Any test using certified eye-tracking systems for that purpose can be adapted using an alternating manual or automated cover to obtain skew deviation measurements. To assure meaningful saccade detection, we recommend a frame rate of 250 Hz.

It has been shown in previous studies that skew deviation varies with upright and supine body position in patients with a vestibular imbalance [21]. In our study, we tested participants solely in an upright sitting position.

We studied only healthy subjects and did not test patients with spontaneous nystagmus. Such vestibular nystagmus is predominately horizontal, however, a mixed torsional and upbeat component in incomplete, superior vestibular neuritis, might bias the clinical assessment. Small vertical eye movements from the underlying nystagmus might be falsely interpreted as vertical skew during the clinical APCT. Physiological skew deviation (< = 0.30° [4]) in healthy participants, however, is usually not discernable.

**Fig. 5:**
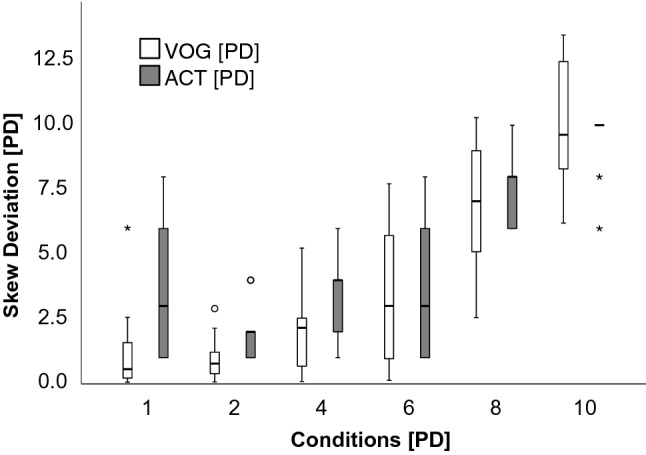
Median prism diopters (PD) obtained by VOG measurement (white boxes) and by the orthoptist using APCT (grey boxes). On the *x*-axis, the different FPs are given in prism diopter

We were not able to induce small skews in all healthy participants due to central adaptation (see condition 1–4 PD in Fig. 5). We, therefore, asked participants to keep their eyes closed before the measurements began to prevent as much as possible prior adaptation and to maximize the success of obtaining a valid skew measurement. Those participants with small induced skews [< 2 PD (1.14°)] were still detectable by the eye tracker, provided that the recorded eye traces were clean and free from artifacts (Fig. 2).

Finally, during the measurements of large skews [10PD (5.7°)], there was selection bias due to a limited ordinal scale from 1 to 10PD (0.57–5.7°) compared to continuous results supplied by the VOG. Our current study investigated artificially induced skew on healthy participants. Thus, it has not yet been tested on patients with pathological skews. Future studies must be validated with patients suffering from conditions such as heterophoria, acute vestibular imbalance or other neuro-ophthalmological ailments affecting the alignment of the eyes.

Additionally, it would be interesting to use this quantitative measurement of skew alongside with other vestibular exams such as oVEMPs, for example, to confirm the involvement of the utricular pathway in transitory skews observed in patients with an acute vestibular syndrome.

### Implications

Skew deviation is part of the ‘HINTS’ protocol first described by Kattah in 2013, [23] this clinical exam is used to distinguish peripheral vestibular pathologies from more dangerous strokes in patients with an acute vestibular syndrome. It consists of a battery of three tests, the head impulse test, the observation of a Nystagmus and the test of Skew [23]. Since VOG is already used for head impulse recordings [24] and nystagmus detection in the ED, [25] we suggest to measure skew deviations with the same VOG equipment as well. Such an approach would not only allow an examiner-independent diagnostic test at the point-of-care, but it would also provide a quantification and automated assessment of vertical skew in the future [26].

## Conclusions

Automated alternate cover test by VOG offers an objective and quantitative skew measurement and proved to be accurate in measuring vertical eye misalignment. Precision was moderate, which mandates a sufficient number of tests per subject. VOG could serve in the future as a quantitative, complementary test for the ACT with prism, thus allowing for its use not only by experts but also by non-experts. Furthermore, it could be used as a future point-of-care diagnostic test for patients with an acute vestibular syndrome in the ED.

## Supplementary Information

Below is the link to the electronic supplementary material.

Supplementary file1 (PDF 250 KB)

## Abbreviations

APCT: Alternated prism cover test
VOG: Video-oculography
HIT: Head impulse test
HINTS: Head impulse nystagmus test of skew
OTR: Ocular tilt reaction
