# Supplementary material for: Automated alternate cover test for ‘HINTS’ assessment: a validation study
Source: Eur Arch Otorhinolaryngol. 2021 Jul 23;279(6):2873–9. doi: 10.1007/s00405-021-06998-w (PMC9072275; doi:10.1007/s00405-021-06998-w)
Supplement: Supplementary file 1 — Supplementary file1 (PDF 250 KB) [file 405_2021_6998_MOESM1_ESM.pdf]

Supplementary Material

Table S1. Mean values and standard deviation per condition

Conditions	N	APCT				VOG			
		Min	Max	Mean [PD]	SD	Min	Max	Mean [PD]	SD
1PD	10	1	8	3.9	2.9	0.1	6.0	1.3	1.8
2PD	10	1	4	2.0	1.2	0.1	2.9	1.0	0.9
4PD	10	1	6	3.5	1.4	0.1	5.2	2.1	1.7
6PD	10	1	8	3.9	2.9	0.1	7.7	3.5	2.6
8PD	10	6	10	7.8	1.5	2.6	10.3	6.8	2.5
10PD	10	6	10	9.4	1.4	6.2	13.5	9.9	2.5

Summary of the mean value of skew measurements for each condition using the APCT and the VOG.

Figure S1. Variance of VOG versus APCT

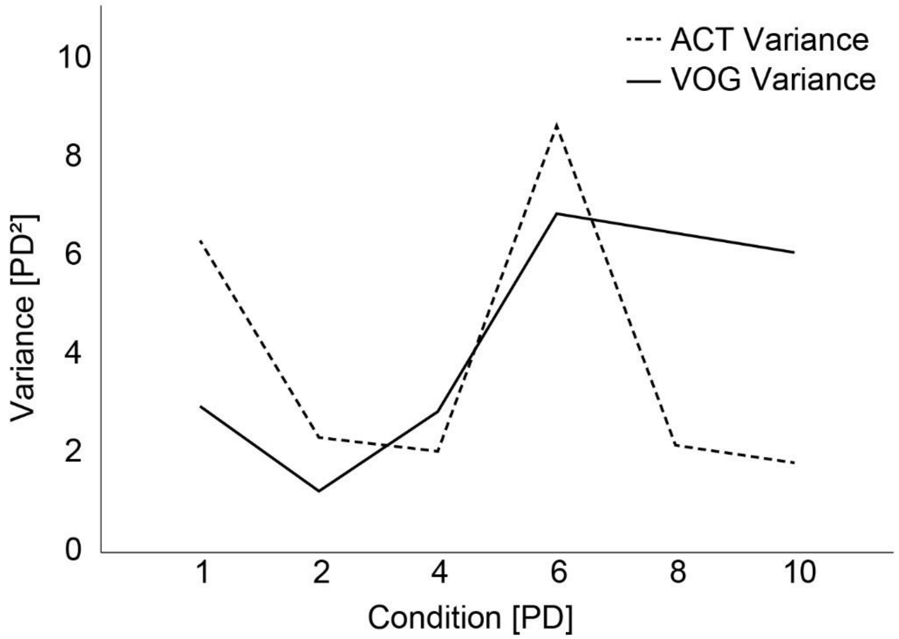

Correlation between the variance of skew assessment and the conditions (Fresnel prism foils). The black continuous line represents the variance of the Skew-VOG test. The non-continuous line represent the variance of the alternating cover test.
